# Supplementary material for: A Rapid Alkalinization Factor-like Peptide EaF82 Impairs Tapetum Degeneration during Pollen Development through Induced ATP Deficiency
Source: Cells. 2023 Jun 4;12(11):1542. doi: 10.3390/cells12111542 (PMC10252199; doi:10.3390/cells12111542)
Supplement: Supplementary file 1 [file cells-12-01542-s001.zip › Supplementary Tables-Figures.pdf]

A rapid alkalinization factor-like peptide EaF82 impairs tapetum degeneration during pollen development through induced ATP deficiency

Chiu-Yueh Hung[1], Farooqahmed S. Kittur[1], Keely N. Wharton[1,a], Makendra L. Umstead[1,b], D'Shawna B. Burwell[1], Martinique Thomas[1,c], Qi Qi[1], Jianhui Zhang[1], Carla E. Oldham[1], Kent O. Burkey[2], Jianjun Chen[3,*] and Jiahua Xie[1,*]

This Supplementary Data file includes:

1. Supplementary Table 1-3, 7-9

Table S1. List of primer sequences used in current study.

Table S2. The Hybrigenics' references of seven selected clones for 1-by-1 assays.

Table S3. The summary of read counts of RNAseq analysis in current study.

Table S7. A subset of FPKM related to tapetum genes.

Table S8. The EaF82 interacting proteins.

Table S9. Summary of interaction matrix and results.

2. Supplementary Figures S1-12

Figure S1. Genetic cassettes used in this study.

Figure S2 Alkalinization assays of inactive EaF82-S as well as a representative of collected pollen grains and tobacco suspension cells.

Figure S3. Tissue specific expression of *EaF82* promoter in Arabidopsis transgenic *EaF82p::EaF82-sGFP* (TA) lines.

Figure S4. Arabidopsis transgenic *EaF82p::EaF82-sGFP* lines (TA-1, -3, -4, and -5).

Figure S5. Immunoblots of Arabidopsis transgenic *EaF82p::EaF82-sGFP (*TA-4) flower proteins against anti-EaF82 and anti-GFP antibodies.

Figure S6. Arabidopsis transgenic *35Sp::EaF82-sGFP* (TB) lines.

Figure S7. RT-qPCR of *GUS* expression levels.

Figure S8. Male gametophyte development of Arabidopsis transgenic *35Sp::EaF82-sGFP* (TB) line.

Figure S9. The numbers of seeds per silique with different length (cm) in Arabidopsis transgenic 35Sp::EaF82 (TC) line.

Figure S10. RNAseq analysis of early developmental flower buds of two independent *35Sp::EaF82* (TC1 and TC2) and vector control (C) transgenic lines.

Figure S11. Solid growth tests on +/- Histidine and +/- 3-AT plates.

Figure S12. A "DomSight" of AKIN10 (AT3G01090) displays the information of bait and prey structural, functional and interaction domains.

Supplementary Tables 4-6 in Excel spreadsheets

Table S4. Common DEGs that both TC-1 and TC-2 increase two-fold compared to the vector control.

Table S5. Common DEGs that both TC-1 and TC-2 decrease two-fold compared to the vector control.

Table S6. A subset of upregulated and downregulated DEGs with FDR<0.05.

Supplementary Data Sets 1-3 in Excel spreadsheets

Data Set S1. The FPKM values for normalized read counts of RNAseq analysis of Arabidopsis transgenic *35Sp::EaF82* (TC) and vector control (C) lines.

Data Set S2. Gene Ontology enrichment analysis of upregulated DEGs.

Data Set S3. Gene Ontology enrichment analysis of downregulated DEGs.

Table S1. List of primer sequences used in current study

AGI ID	gene name	Forward sequence (5'-3')	Reverse sequence (5'-3')
**At1G19890**	*MGH3*	AGGCAGCCGAAGCATACTTG	ACCCTTTTGGCGTGAATCG
**At1G19960**	*AT1G19960*	CGGCGGCTCCTTTGG	CCACACTTGCTTCCTCACCTTT
**At1G21000**	*AT1G21000*	ACGGTCCCAATGGTGATCA	GGAGAGAAACTAAAACCACCTTCATC
**At1G24520**	*BCP1*	CGATGACGATGCAGCTCCTA	TAGAGGACCAGCCACTGCAA
**At1G28270**	*RALFL4*	CAATGCAACCTACCCTTTAACCA	GTCTTCCCCGATGCAACCT
**At1G29140**	*AT1G29140*	TGAGTGCAAGGGTCGTGAGA	TTGTCGGTCACGGCTTCTTT
**At1G35490**	*AT1G35490*	CATCGAGCGCGTTTGAGA	TTGTAGCACTTGGATGGTCCTTT
**At1G61563**	*RALFL8*	CCCACCCTAACACCTGCAA	CTTTGGACCTGTTTCACGATGG
**At1G61566**	*RALFL9*	CCCACCCTAACACCTGCAA	ATCTTCTCGCATCCTCTCTGGTA
**At1G80660**	*HA9*	TGGCACCGTGTCAGCAAA	CTTGCATCCTCCCGAAGGT
**At2G07040**	*PRK2A*	GCGCGCAAGAGCTCATG	TTGGTCACACGGCTTTGTTTT
**At2G07560**	*HA6*	GGGTTGCACGTCAGAGAGTT	TTGGAGGGTCAAAGAGTGGC
**At2G18470**	*PERK4*	GCAAGAAATGGCTCGAATGG	CGCCCCGAATGACGAAT
**At2G21480**	*AT2G21480*	TGAACGGTGTCGAGGTCTTG	ATCCCCTGCTTCCCCATACT
**At2G22055**	*RALFL15*	CAAAGCCCACCCTAACACCT	ACCTGTATCACGACGGCAAC
**At2G33775**	*RALFL19*	TGTTGATCCTTGGCCTCTTGA	GGGTCCATGTGGCATTGG
**At2G36080**	*ABS2*	GGAGAGCCAAAGAGGCAACT	CCGAATCTAGCTGGCACTCC
**At2G45660**	*AGL20*	ACTCTTGGGAGAAGGCATAGGAA	TCAAGCTGTTGCTCAATCTGTTG
**At3G08560**	*VHA-E2*	AAGCCAAAGTCGGGTCTCCTA	AGGAGGTGGAGGGAGGAAGA
**At3G25165**	*VHA-E2*	GGACGAGTTGAGGCTAACGATAA	GGAGCATTAGGACGCAAGCA
**At3G42640**	*HA8*	GGGGAGCAAGAGGCTTCAAT	CCTTCGAGGAGACGAGCATC
**At4G10603**	*AT4G10603*	CCGTTGTTCGGCTAGGTGTAAG	CATTGCCTTGCCCCCTTTA
**At4G14020**	*AT4G14020*	ACATTTACCGAAAACGACGAAGAA	TTCTTTCACCCACGTGTTATTCA
**At4G24540**	*AGL24*	ACGTTGGAAAGGGCAAAACTG	TTTGTGGTCACCGACTCTGTCT
**At4G35900**	*FD*	GCAAGACTCAAGAGACAACAAG	CAAAATGGAGCTGTGGAAGAC
**At4G39110**	*AT4G39110*	AGGGCAAAGCAGAAGAGACC	TTCGTTGGTGGTGACTGAGG
**At5G17480**	*PC1*	ATGGCTGATGCAACGGAGAA	GAGCTTCTTCGAGTTCGGCT
**At5G28680**	*ANX2*	AGCAGTTTTTGTCTTGTTGGGG	CTGGAGGCTTGTTTCTGCAA
**At5G45880**	*AT5G45880*	CTTGCCGTGTCCAATTCGTT	TTCTGCTCCTGCACTCCAAC
**At5G57350**	*HA3*	TTCCTGCTGATGCACGTCTT	TTCCCCTGGACCTTTCGTTG
**At5G62165**	*AGL42*	CCAGCAATCACGACTCACAAAT	TTGTTATCATGTGGCTTGCTTCTT
	EaF82	GCATGTGGTGTCACTCCTCTTTAT	ATCTTCTTCCGCAGTCCCAAA
	NPTII	AAGATGGATTGCACGCAGGTTC	ACGGGTAGCCAACGCTATGTC
	GUS2	TGGCCTGGCAGGAGAAACT	ACGTATCCACGCCGTATTCG

Table S2. The Hybrigenics' references of seven selected clones for 1-by-1 assays

ATMB_RP1_hgx4998v1_pB27_A-237 ABCF4 [402-557]*
ATMB_RP1_hgx4998v1_pB27_A-240 ALATS [36-282]
ATMB_RP1_hgx4998v1_pB27_A-66 FKBP-like peptidyl-prolyl cis-trans isomerase family protein [43-190]
ATMB_RP1_hgx4998v1_pB27_A-262 PAPP2C [177-287]
ATMB_RP1_hgx4998v1_pB27_A-94 TCH4 [24-178]

ATMB_RP1_hgx4998v1_pB27_A-125 AKIN10 [422-535]
ATMB_RP1_hgx4998v1_pB27_A-157 SYTA [309-536]

*The amino acid residues of proteins been identified in prey fragments are indicated in brackets. The prey fragments that do not stop with the natural stop codon of the protein have a short additional peptide at the C-terminal end which comes from the SfiI restriction side, being used for cloning of the library fragments.

Table S3. The summary of read counts of RNAseq analysis in current study

Samples ID	Read	Read length bp	Index	Number of Reads
**C1**	SE	76	CTGAAGCT+AGGCTATA	19,858,927
**C2**	SE	76	TAATGCGC+AGGCTATA	18,609,048
**C3**	SE	76	CGGCTATG+AGGCTATA	18,331,258
**TC1-1**	SE	76	CTGAAGCT+GCCTCTAT	20,035,018
**TC1-2**	SE	76	TAATGCGC+GCCTCTAT	19,216,782
**TC1-3**	SE	76	CGGCTATG+GCCTCTAT	20,693,114
**TC2-1**	SE	76	CTGAAGCT+AGGATAGG	23,037,461
**TC2-2**	SE	76	TAATGCGC+AGGATAGG	21,102,277
**TC2-3**	SE	76	CGGCTATG+AGGATAGG	21,339,999

Table S7. A subset of FPKM related to tapetum genes.

Gene id	Gene short name	locus	length	FPKM_C1	FPKM_C2	FPKM_C3	FPKM_TC1-1	FPKM_TC1-2	FPKM_TC1-3	FPKM_TC2-1	FPKM_TC2-2	FPKM_TC2-3
**Tapetum-related**												
**AT5G07280**	**EMS1**/EXS	5:2284829-2288855	4.03E+03	12.94	13.09	15.33	16.05	12.87	13.77	15.54	14.20	14.92
**AT4G24972**	**TPD1**	4:12837352-12840230	1.34E+03	6.83	4.42	6.49	3.74	5.79	5.30	5.00	4.87	4.98
**AT1G34210**	**SERK2**	1:12458563-12462905	2554	9.38	10.01	9.56	10.24	10.99	7.75	10.13	6.65	9.18
**AT1G34210**	**SERK2**	1:12458589-12462752	2267	6.15	5.44	8.14	5.18	5.28	4.95	5.58	10.70	6.98
**AT1G71830**	**SERK1**	1:27018157-27022117	2570	12.46	13.51	14.42	14.26	14.75	14.41	15.81	14.42	15.47
**AT3G28470**	**TDF1**/MYB35	3:10674507-10675724	1025	1.37	2.02	1.91	2.62	0.97	1.34	2.08	1.48	2.71
**AT2G16910**	**AMS**	2:7331720-7334254	1893	33.39	33.40	45.57	48.79	29.50	33.23	47.66	35.97	44.85
**AT5G56110**	**MYB188/MYB103/MYB80**	5:22719190-22720664	963	3.39	2.56	4.60	2.55	1.20	2.07	2.87	1.67	3.67
**AT5G22260**	**MS1**	5:7367634-7370295	2.19E+03	2.59	2.50	3.25	3.28	2.23	1.66	3.51	2.43	3.18

**AT4G21330**	**DYT1**	4:11349921-11350694	624	0.13	0.68	0.28	1.92	0.27	0.87	1.58	1.09	1.32
**AT4G20900**	**MS5**	4:11184102-11185844	1353	0.00	0.00	0.00	0.00	0.00	0.00	0.00	0.00	0.00
**AT4G20900**	**MS5**	4:11184102-11185844	1305	1.17	0.73	1.80	1.81	0.57	1.00	1.29	1.38	1.47
**AT3G51280**	**MS5-like**	3:19037139-19039048	1649	20.20	27.64	26.55	38.31	41.52	32.95	50.05	41.90	41.58
**AT3G11980**	**MS2**/FAR2	3:3814230-3817117	2117	51.40	33.34	54.30	60.38	45.99	35.70	64.57	52.92	69.76
**AT4G33790**	**MS2-like**/FAR3	4:16204095-16207956	1956	0.73	0.49	1.02	1.31	0.54	1.30	0.22	1.51	0.75
**AT4G33790**	**MS2-like**/FAR3	4:16204095-16207956	1776	39.84	34.90	39.31	37.02	40.67	27.61	28.08	28.10	32.53
**AT4G27330**	**SPL/NZZ**	4:13682077-13683594	1329	6.96	6.80	9.52	5.98	3.94	6.38	5.31	5.33	5.67
**AT5G50260**	**CEP1***	5:20455316-20457068	1580	**6.55**	**2.78**	**5.29**	**0.27**	**0.37**	**0.53**	**0.28**	**0.15**	**0.22**
**AT2G01570**	**RGA**	2:255246-257568	2.32E+03	33.43	39.86	40.28	38.23	40.33	38.79	37.86	40.58	43.17
**AT4G02780**	**GA1**	4:1237670-1244822	2675	0.41	0.62	0.57	0.59	0.64	0.38	0.56	0.62	0.70
**AT1G14920**	**GAI**	1:5148981-5151415	2434	19.30	23.02	21.23	25.44	28.62	24.79	25.76	24.24	29.28
**AT5G06100**	**MYB33**	5:1837834-1840706	2266	2.96	2.58	4.12	3.55	3.76	6.08	6.83	5.50	2.60
**AT5G06100**	**MYB33**	5:1837834-1840727	2206	8.33	7.79	6.27	8.20	6.43	4.91	4.10	5.46	8.46
**AT5G06100**	**MYB33**	5:1837895-1840701	2537	0.00	0.00	0.02	0.00	0.00	0.00	0.00	0.00	0.13
**AT5G06100**	**MYB33**	5:1837913-1840159	1741	0.00	0.00	0.00	0.00	0.21	0.00	0.23	0.39	0.73
**AT5G06100**	**MYB33**	5:1837913-1840706	2443	0.20	0.00	1.79	0.62	0.72	1.02	0.36	0.40	0.04
**AT5G06110**	**MYB33**	5:1840754-1843872	2554	28.83	19.05	32.29	36.99	33.59	28.20	35.01	26.77	31.72
**AT3G11440**	**MYB65**	3:3602035-3605172	2.23E+03	0.00	0.25	0.26	0.00	0.00	0.00	0.00	0.00	0.00
**AT3G11440**	**MYB65**	3:3602035-3605172	2140	0.00	1.94	0.63	0.42	1.31	0.00	0.37	0.00	0.78
**AT3G11440**	**MYB65**	3:3602348-3605110	2089	0.00	0.00	0.00	0.00	0.00	0.00	0.00	0.00	0.00
**AT3G11440**	**MYB65**	3:3602883-3605104	2009	3.24	0.53	2.97	2.36	1.68	3.34	3.44	3.07	2.28

*: The gene is in the downregulated DEG list.

Table S8. The EaF82 interacting proteins

	name	ATG	# clones	score
	Arabidopsis thaliana - BIP1	AT5G28540	16	A
**p**	Arabidopsis thaliana - ALATS	AT1G50200	10	A
**p**	Arabidopsis thaliana - SYTA	AT2G20990	10	A

	Arabidopsis thaliana - F17H15.1	AT2G25970	7	A
	Arabidopsis thaliana - MNJ7.18	AT5G47590	7	A
	Arabidopsis thaliana - SOX	AT3G01910	7	B
	Arabidopsis thaliana - F14O23.22	AT1G71840	5	B
	Arabidopsis thaliana - HSC70-1	AT5G02500	5	B
	Arabidopsis thaliana - F5F19.6	AT1G52000	4	B
	Arabidopsis thaliana - FP3	AT5G63530	4	B
**p**	Arabidopsis thaliana - PAPP2C	AT1G22280	4	B
**p**	Arabidopsis thaliana - ABCF4	AT3G54540	3	C
	Arabidopsis thaliana - LOS2	AT2G36530	3	C
	Arabidopsis thaliana - SPDS2	AT1G70310	3	C
	Arabidopsis thaliana - emb1507	AT1G20960	3	C
	Arabidopsis thaliana - heat shock protein 70-3	AT3G09440	3	C
	Arabidopsis thaliana - MFB16.14	AT5G50740	5	D
	Arabidopsis thaliana - PATL1	AT1G72150	5	D
	Arabidopsis thaliana - ACT2	AT3G18780	2	D
	Arabidopsis thaliana - AMY3	AT1G69830	2	D
	Arabidopsis thaliana - Carbohydratebinding-like fold	AT3G62360	2	D
	Arabidopsis thaliana - F13M22.2	AT2G37520	2	D
**p**	Arabidopsis thaliana - FKBP-like	AT3G55520	2	D
	Arabidopsis thaliana Translation elongation factor EFG/EF2 protein (SCO1)	AT1G62750	2	D
	Arabidopsis thaliana - HIP1	AT4G22670	2	D
	Arabidopsis thaliana - IDM2	AT1G54840	2	D
	Arabidopsis thaliana - MYC3	AT5G46760	2	D
	Arabidopsis thaliana - SK5	AT3G60020	2	D
	Arabidopsis thaliana - SRC2	AT1G09070	2	D
**p**	Arabidopsis thaliana - TCH4	AT5G57560	2	D
	Arabidopsis thaliana - clathrin	AT5G05010	2	D
**p**	Arabidopsis thaliana - AKIN10	AT3G01090	1	D
	Arabidopsis thaliana - DRH1	AT3G01540	1	D
	Arabidopsis thaliana - F18O14.16	AT1G19400	1	D
	Arabidopsis thaliana - HSP91	AT1G79930	1	D
	Arabidopsis thaliana - MBP1	AT1G52040	1	D
	Arabidopsis thaliana - MDJ22.18	AT5G22760	1	D
	Arabidopsis thaliana - MJJ3.25	AT5G05830	1	D
	Arabidopsis thaliana - MPK17.2	AT5G36740	1	D
	Arabidopsis thaliana - PBB2	AT5G40580	1	D
	Arabidopsis thaliana - Phox3	AT5G20360	1	D
	Arabidopsis thaliana - SGT1A	AT4G23570	1	D
	Arabidopsis thaliana - T19D16.19	AT1G10890	1	D
	Arabidopsis thaliana - T1K7.11	AT1G26520	1	D
	Arabidopsis thaliana - cpHsc70-1	AT4G24280	1	D
	Arabidopsis thaliana - hypothetical protein	AT3G59430	1	D

Table S9. Summary of interaction matrix and results

Interaction Matrix					Selection Medium					
N# Interaction*	Type	Bait	Prey	Prey clone reference	DO-2	DO-3	DO-3 +	DO-3 +	DO-3 +	DO-3 +

							1mM 3-AT	5mM 3-AT	10mM 3-AT	50mM 3-AT
1	Hybrigenics' positive control	SMAD	SMURF	/	+	+	+	+	+	+
2	Negative control	F82	pP7ø	/	+	-	-	-	-	-
3	Negative control	pB27ø	ABCF4	pB27_A-237	+	-	-	-	-	-
4	Interaction	F82	ABCF4	pB27_A-237	+	+	+	+	+/-	-
5	Negative control	pB27ø	ALATS	pB27_A-240	+	-	-	-	-	-
6	Interaction	F82	ALATS	pB27_A-240	+	+	+	-	-	-
7	Negative control	pB27ø	FKBP_like peptidyl-prolyl cis-trans isomerase family protein	pB27_A-66	+	-	-	-	-	-
8	Interaction	F82	FKBP_like peptidyl-prolyl cis-trans isomerase family protein	pB27_A-66	+	+	+	+/-	-	-
9	Negative control	pB27ø	PAPP2C	pB27_A-262	+	-	-	-	-	-
10	Interaction	F82	PAPP2C	pB27_A-262	+	+	+	+	+	
11	Negative control	pB27ø	TCH4	pB27_A-94	+	-	-	-	-	-
12	Interaction	F82	TCH4	pB27_A-94	+	+	+	-	-	-
13	Negative control	pB27ø	AKIN10	pB27_A-125	+	-	-	-	-	-
14	Interaction	F82	AKIN10	pB27_A-125	+	+	+	+	+/-	-
15	Negative control	pB27ø	SYTA	pB27_A-157	+	-	-	-	-	-
16	Interaction	F82	SYTA	pB27_A-157	+	+	+	-	-	-

*A single clone of each negative control is spotted in duplicates. pB27: LexA DNA Binding Domain (DBD) vector (LexA-bait); pB27ø: empty pB27 vector; pP7/pP6: Gal4 Activation Domain (AD) vector (AD-prey); pP7ø: empty pP7 vector; DO-2: selective medium without tryptophan and leucine; DO-3: selective medium without tryptophan, leucine and histidine; 3-AT: 3-aminotriazole.

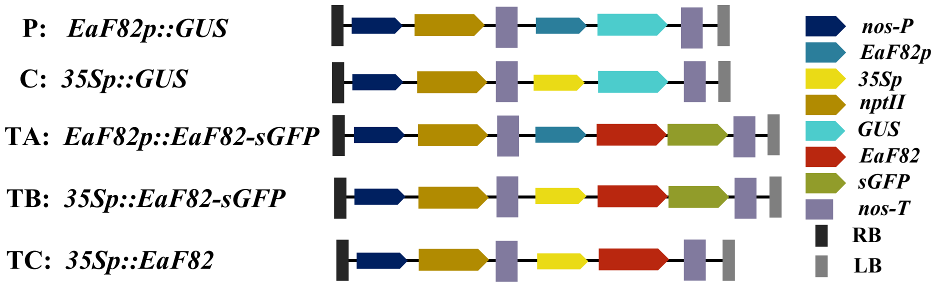

Figure S1 Genetic cassettes used in this study. *EaF82p: EaF82* promoter; *35Sp*: CaMV *35S* promoter; *sGFP: Green Fluorescent Protein* (S65T)*; GUS*: *uidA*; *nptII: neomycin phosphotransferase II*; *nos-P: nopaline synthase* promoter; *nos*-T: *nos* terminator; RB: right border; LB, left border.

a

b

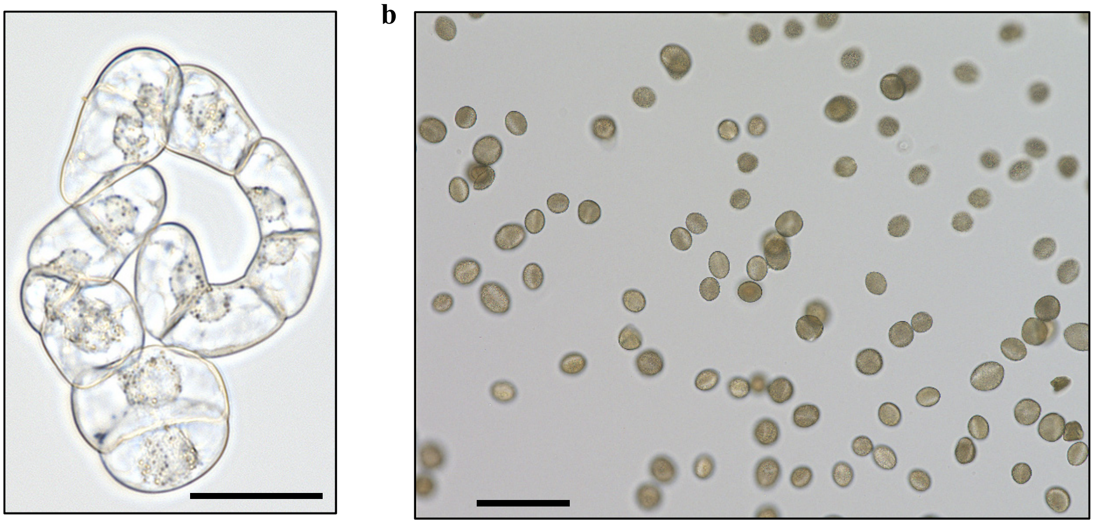

c

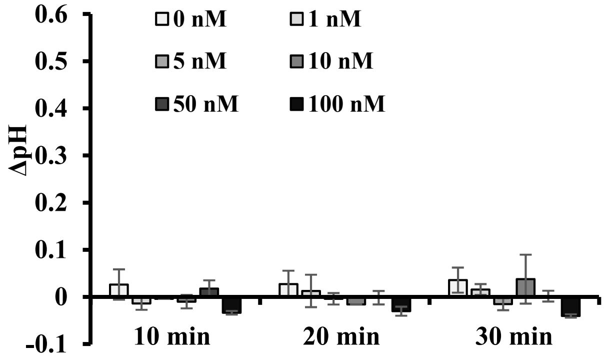

Figure S2 Alkalinization assays of inactive EaF82-S as well as a representative of collected pollen grains and tobacco suspension cells. **a** A representative of tobacco suspension cells used for alkalinization assay. Bar=50μm. **b** A representative of collected pollen in HEPES potassium salt buffer for protein isolation. Bar=100μm. **c** Alkalinization assays of inactive EaF82-S. Data plotted is the concentration curve. The pH changes (Δ pH) were calculated as measured pH value after applying inactive EaF82-S for 10, 20 and 30 min minus the pH value at 0 min at each concentration from 0 to 100nM. The data plotted were the average of three independent experiments ± SD.

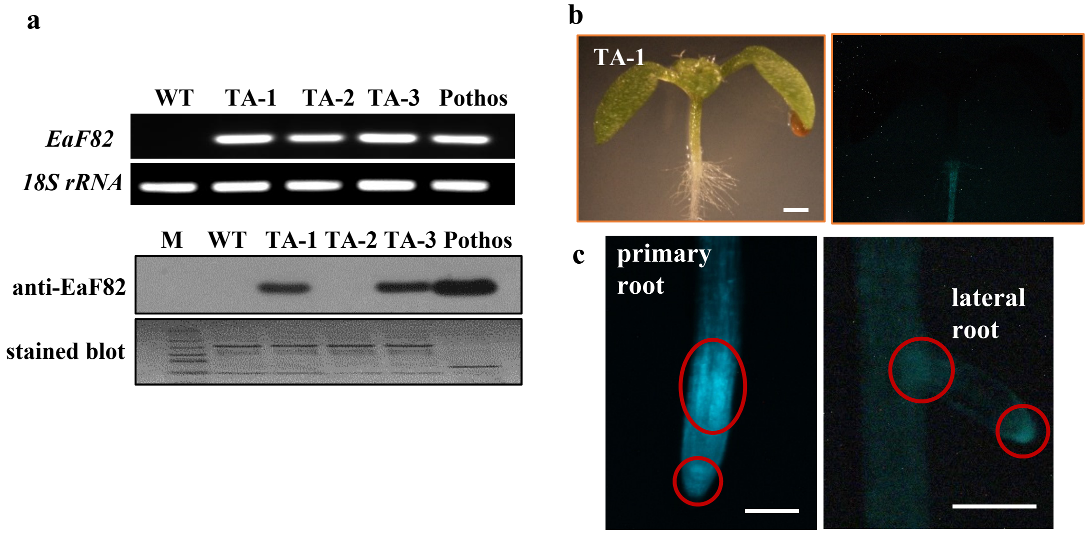

Figure S3 Tissue specific expression of *EaF82* promoter in Arabidopsis transgenic *EaF82p::EaF82-sGFP* (TA) lines. **a** RT-PCR (upper panel) and immunoblot (lower panel) of three 7-day old subline seedlings (TA-1, 2, and 3). PCR was performed using primer pairs specific to *EaF82* and *18S rRNA* gene (as internal control). Stained blot shows protein loading. *EaF82* originated from 'Golden Pothos' was used as a positive control. **b** 10-day old light grown seedlings (left) and the detected GFP signals in roots (right). Bar = 1mm. **c** GFP signals detected in primary and lateral roots are circulated in red. Bar = 500 µm.

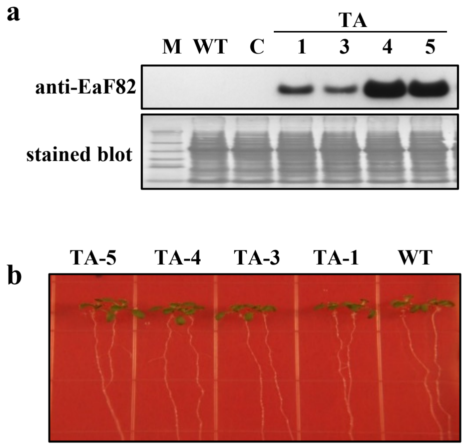

Figure S4 Arabidopsis transgenic *EaF82p::EaF82-sGFP* lines (TA-1, -3, -4, and -5). **a** Immunoblot of seedling proteins against anti-EaF82. C: vector control. WT: wild-type. M: protein size marker. **b** 10-day old seedlings germinated on MS medium show normal growth as WT.

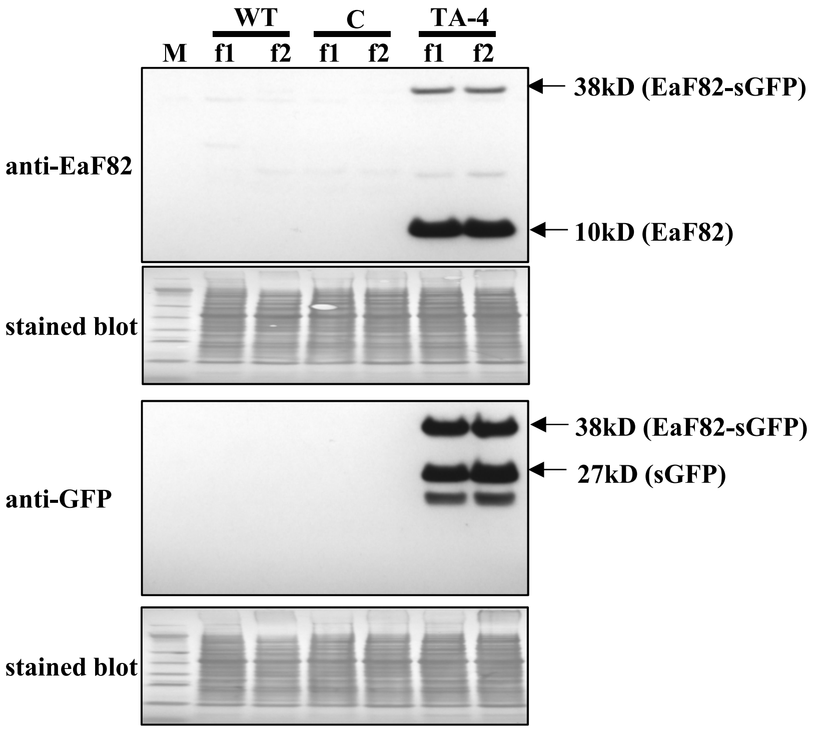

Figure S5 Immunoblots of Arabidopsis transgenic *EaF82p::EaF82-sGFP (*TA-4) flower proteins against anti-EaF82 and anti-GFP antibodies. Stained blots show protein loading. C: vector control. WT: wild-type. M: protein size marker. f1 and f2: two independent pools of flower tissues.

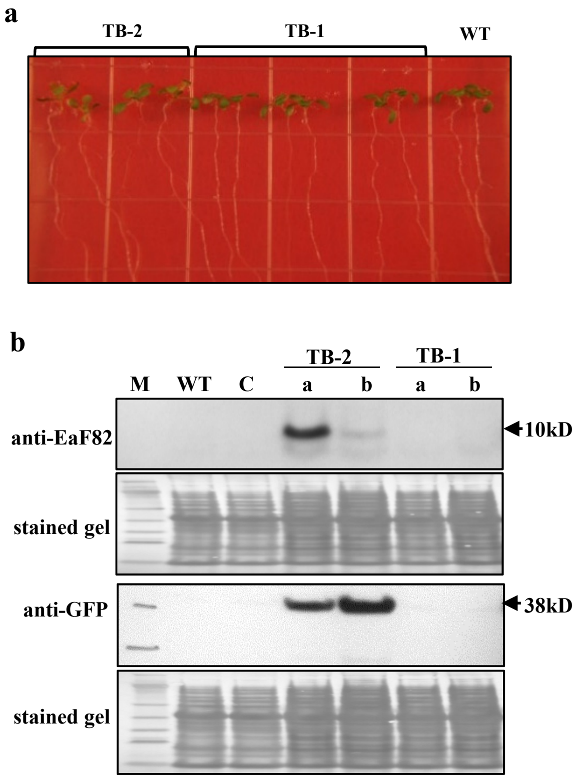

Figure S6 Arabidopsis transgenic *35Sp::EaF82-sGFP* (TB) lines. **a** 10-day old seedlings germinated on MS medium show normal growth as wild-type (WT). **b** Immunoblot of seedling proteins against anti-EaF82 and anti-GFP. TB-1 and -2 are two independent lines, while a, and b are two sublines.

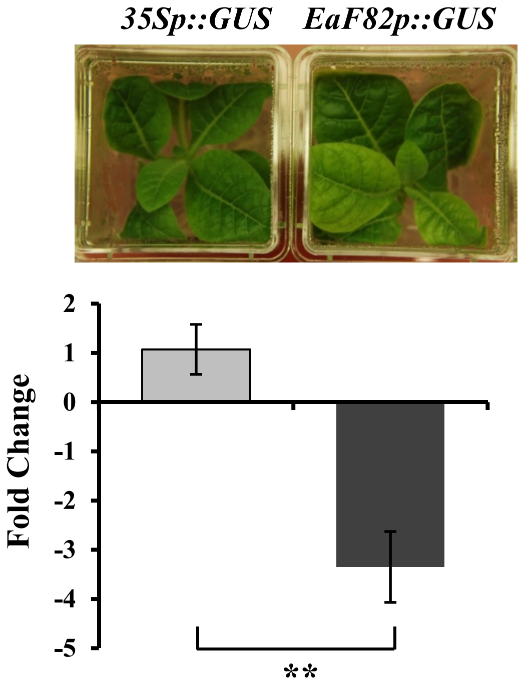

Figure S7 RT-qPCR of *GUS* expression levels. Transgenic tobacco plants carrying *GUS* driven by CaMV *35S* promoter (*35Sp:GUS*) and *EaF82* promoter (*EaF82p::GUS*), respectively (upper panel). GUS expression levels of each transgenic line were first normalized with their internal *18S rRNA* expression level. The fold changes were then calculated as *EaF82p::GUS* comparing to *35Sp::GUS* (as 1). Data plotted are the average of fold changes in three biological replicates ± SD. ** $P<0.01$.

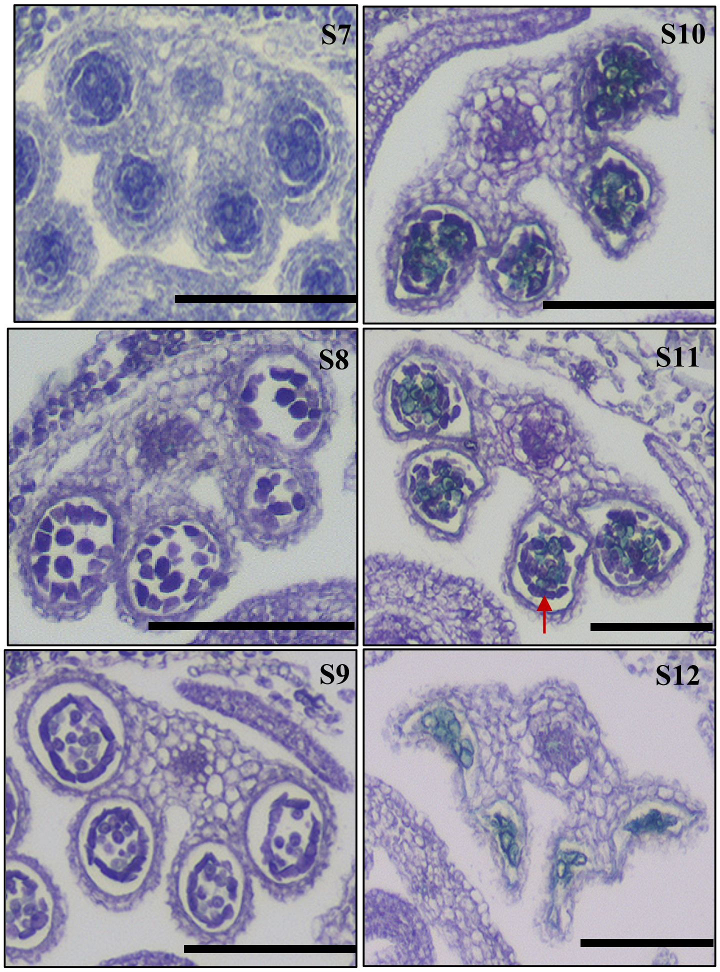

Figure S8 Male gametophyte development of Arabidopsis transgenic *35Sp::EaF82-sGFP* (TB) line. The stages S7 to S12 of a second independent TB flower cluster. The delayed degeneration of tapetum at stage S11 is indicated in red arrow. Underdeveloped pollen at stage S12 are stained in light green. Bar = 100 µm.

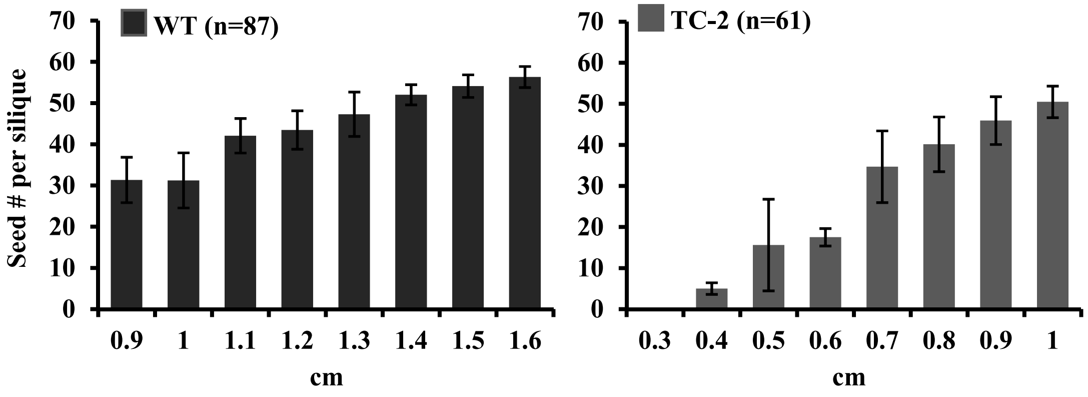

Figure S9 The numbers of seeds per silique with different length (cm) in Arabidopsis transgenic 35Sp::EaF82 (TC) line. Data plotted are the average ± SD. WT: wild-type; n: number of siliques.

a

Genomes	Total listed	Non-detected	% of non-detected
**nuclear**	**32541**	**4382**	**13.5**
**mitochondrial**	**152**	**13**	**8.6**
**plastid**	**133**	**16**	**12.0**

b

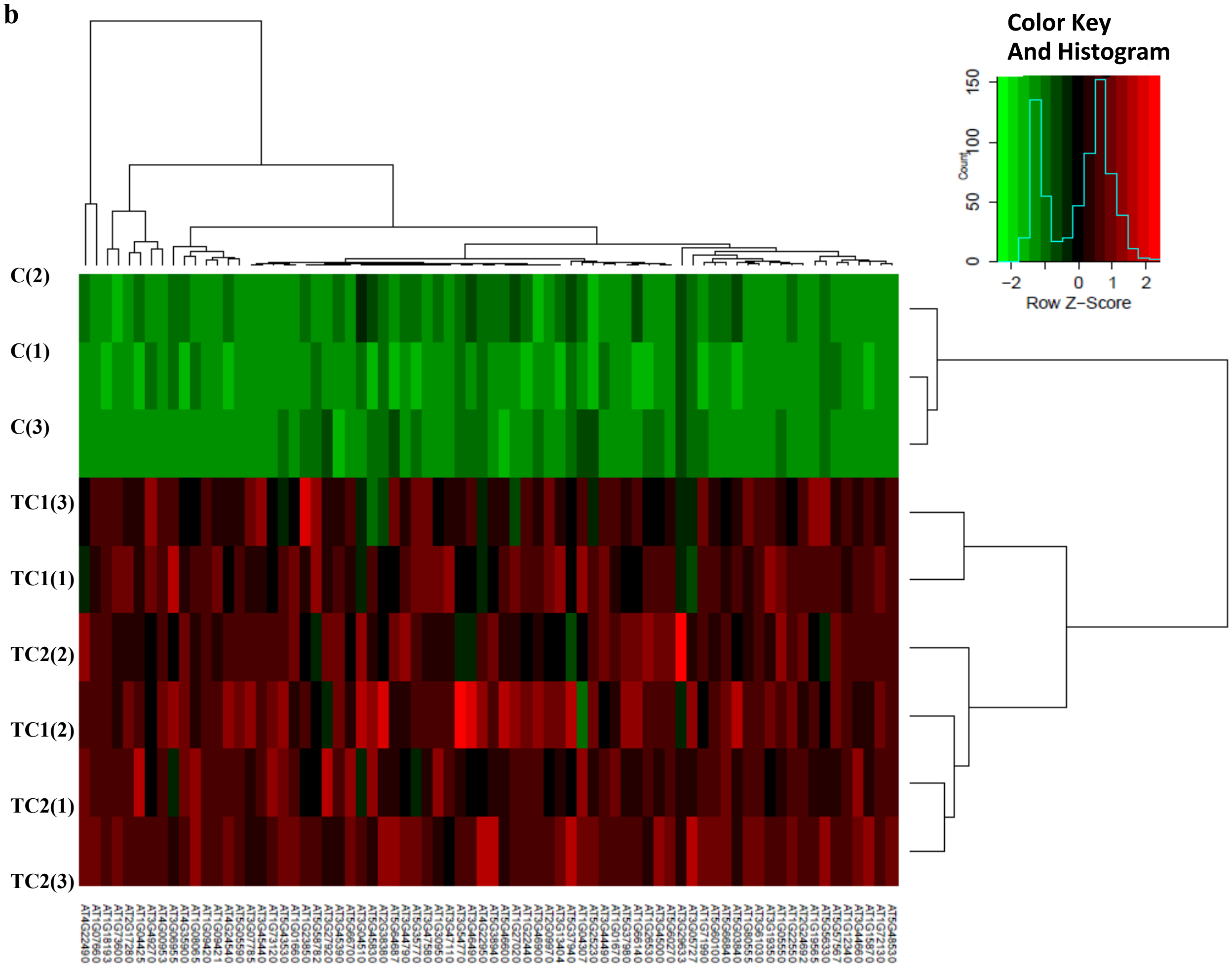

Figure S10 RNAseq analysis of early developmental flower buds of two independent *35Sp::EaF82* (TC1 and TC2) and vector control (C) transgenic lines. **a** The numbers of FPKM_gene listed in Data Set 1. **b** Heat map of hierarchical cluster analysis on DEGs of TC1 and TC2 both with $\log_2(FC) \leq -1.5$. Three biological replicates are indicated as TC1 (1, 2 and 3), TC2 (1, 2 and 3) and C (1, 2 and 3).

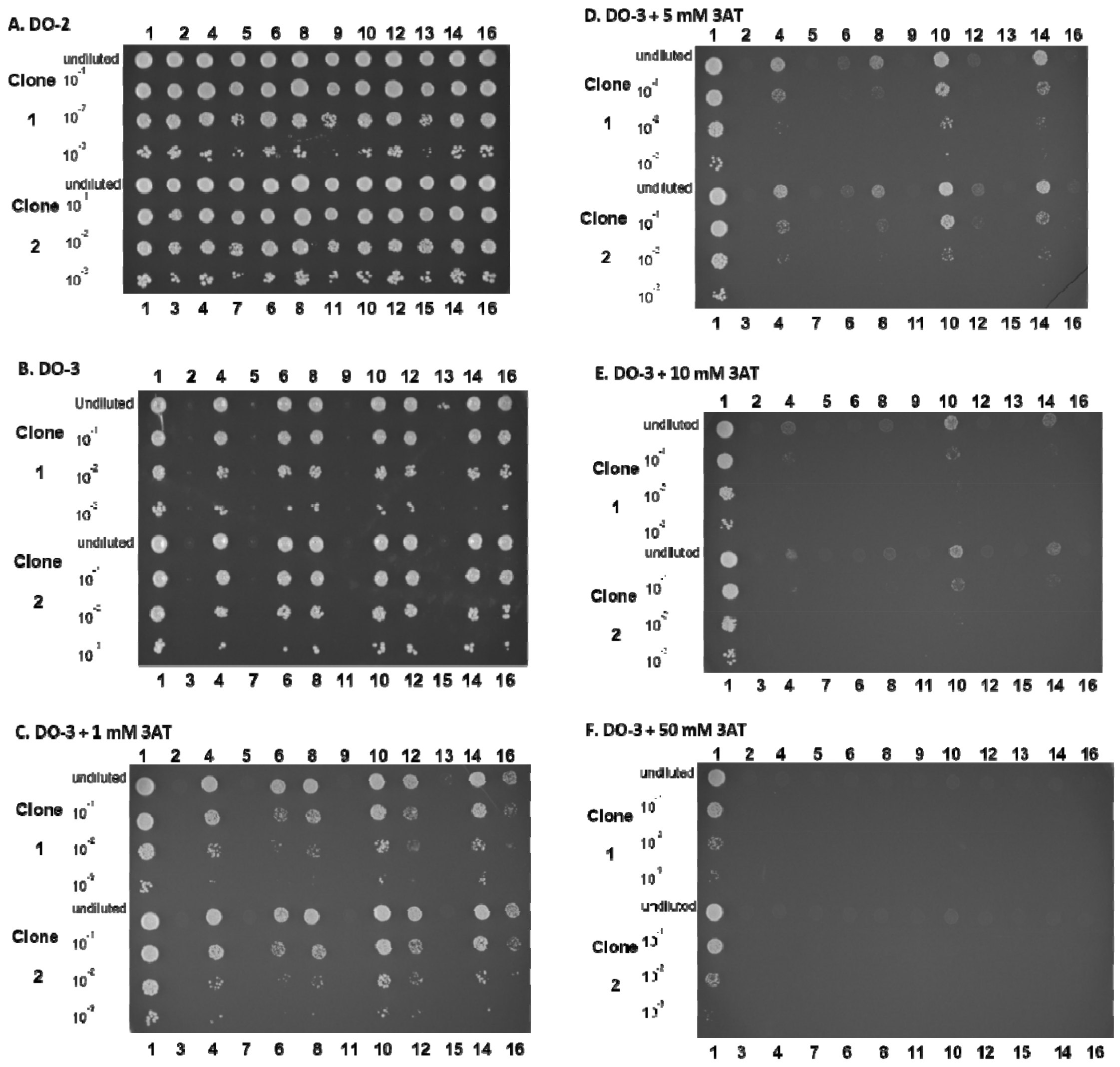

Figure S11 Solid growth tests on +/- Histidine and +/- 3-AT plates. **A** DO-2, **B** DO-3, **C** DO-3 + 1 mM AT, **D** DO-3 + 5 mM AT, **E** DO-3 + 10 mM AT, and **F** DO-3 + 50 mM AT. Detailed information of the interacting #1-16 are listed in Supplementary Table 8.

DomSight index

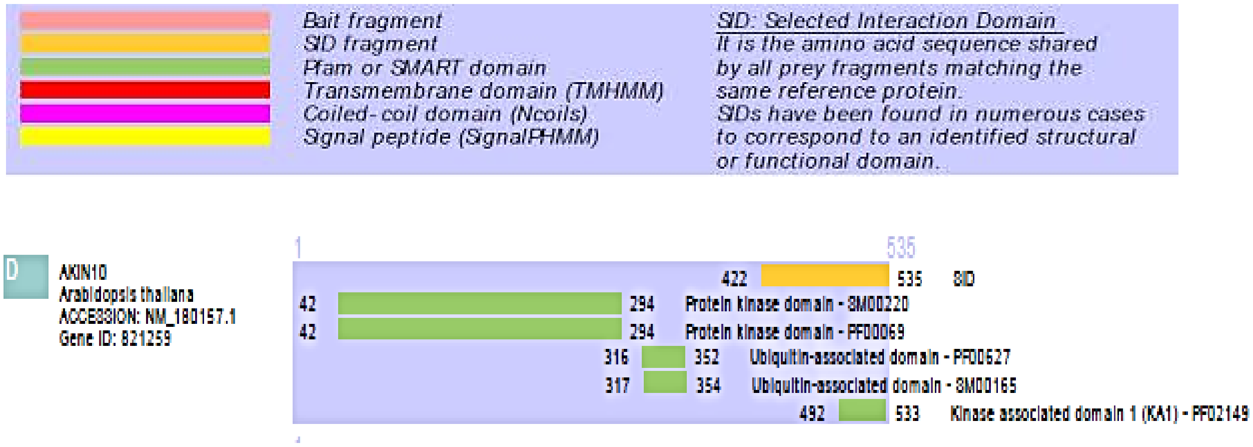

Figure S12 A "DomSight" of AKIN10 (AT3G01090) displays the information of bait and prey structural, functional and interaction domains. The SID domain (amino acid residues 422-535) contains kinase associated domain 1 (KA1).
